# Excission of the Larynx

**Published:** 1874-05

**Authors:** 


*ST. LOUIS MEDICAL AND SURGICAL JOURNAL—March*, 1874.

Excision of the Larynx.—Dr. T. L. Stuever, of Vienna, writes to Dr. E. Montgomery, of this city, that a short time ago Professor Billroth extirpated the greater portion of the larynx in a patient at the Vienna Hospital. It seems this organ was very extensively involved in carcinomatous degeneration, and with the hope of saving the patient's life, this very difficult and dangerous operation was undertaken and successfully performed. Dr. Stuever, who is assistant to Dr. Stork, the celebrated laryngoscopist, writes that the latter has invented an apparatus to supply the place of the excised larynx, by which the patient is enabled to speak distinctly, so as to be audible at a distance of ten feet, but of course without much modulation of voice. Dr. Stuever also mentions that he can breathe and swallow with perfect ease and composure, and is in every way in a most promising condition for complete recovery.

